# Adverse Childhood Experiences, Maternal/Fetal Attachment, and Maternal Mental Health

**DOI:** 10.1089/whr.2020.0085

**Published:** 2020-12-07

**Authors:** Jennifer Hinesley, Ananda Amstadter, Aradhana Sood, Robert A. Perera, Ronald Ramus, Susan Kornstein

**Affiliations:** ^1^ Department of Family Medicine and Population Health, Virginia Commonwealth University, Richmond, Virginia, USA.; ^2^ Department of Psychiatry, Virginia Commonwealth University, Richmond, Virginia, USA.; ^3^ Department of Biostatistics, and Virginia Commonwealth University, Richmond, Virginia, USA.; ^4^ Department of Obstetrics and Gynecology, Virginia Commonwealth University, Richmond, Virginia, USA.

**Keywords:** adverse experiences, parenting, attachment

## Abstract

***Background:*** This pilot study investigated the potential impact of exposure to childhood adversity on variables known to be related to posttraumatic stress (including attachment, mental health, and perceived stress) in a clinic sample of pregnant women.

***Materials and Methods:*** Participants consisted of 101 pregnant women recruited from the Virginia Commonwealth University Health System in Richmond, VA. All participants completed the Adverse Childhood Experience (ACE) questionnaire, Parental Bonding Instrument, Maternal Fetal Attachment Scale, Posttraumatic Stress Disorder (PTSD) Checklist, Symptom Checklist, and the Perceived Stress Scale.

***Results:*** Increased exposure to ACEs was negatively associated with retrospective report of viewing one's mother and father as caring and involved. ACE exposure was a statistically significant predictor of viewing one's mother and father as intrusive and controlling. ACEs were positively associated with self-reported PTSD symptoms, depressive and anxious symptomatology, and perceived stress. No direct effect of adverse childhood events on maternal/fetal attachment was found.

***Conclusions:*** ACE associations are discussed in terms of study methodology and needs for future research. Providers may consider incorporating the ACE questionnaire to identify exposure to childhood adversity and events that may increase an individual's risk for toxic stress and negative health outcomes.

## Introduction

Since the publication of the Adverse Childhood Experiences (ACEs) study,^1^ there has been growing and consistent evidence that exposure to childhood maltreatment (*e.g.*, physical abuse, sexual abuse, and neglect) and family household disturbance (*e.g.*, death of a parent, exposure to parental substance abuse, or criminal activity) is associated with an array of physical and mental health problems. Indeed, individuals who have experienced childhood adversity exhibit higher rates of depression, anxiety, substance abuse, eating disorders, and suicidality,^2^ as well as poorer response to psychosocial and pharmacological treatment.^3^ Moreover, it has been estimated that childhood adversity accounts for 26% to 32% of the risk for all adolescent and adulthood psychiatric disorders attributed at a population level.^4^ The effects of exposure to adversity extend beyond psychiatric impacts to include diminished cognitive functioning and compromised health status.^5^ Individuals who have experienced childhood adversity are more likely to experience immune disorders, cardiovascular disease, and cancer,^1^ and to have premature mortality.^6^ Such individuals are also at increased risk of experiencing interpersonal violence throughout development into adulthood, either as a victim or perpetrator, and to have a lower quality of life. As such, the long-term effects of childhood adversity are significant, pervasive, and represent a critical public health concern.

The transition to parenthood is a unique developmental phase that constitutes a period of stressful and sometimes maladaptive change for a significant proportion of new parents. Caring for an infant or young child can be taxing among the healthiest of parents, particularly in times of high stress. Adults who have experienced adverse events in childhood, such as abuse and neglect, are at higher risk for experiencing parenting challenges on a day-to-day basis. These adults are also at higher risk for reporting greater levels of parenting stress, which has been associated with problematic parenting and poor developmental outcomes in children.^7^ This is a particularly important area of study in light of research positing an intergenerational component in the transmission of trauma.^8^ Such research is also needed in light of early childhood maltreatment statistics indicating that the highest rates of child abuse and fatalities occur as the result of physical abuse during the first 5 years of life.^9^ Among these children, four-fifths (80.3%) of perpetrators were parents, further underscoring the converging evidence that young children's early development is significantly impacted by the mental health of their parents. Therefore, pregnant women who have experienced adverse experiences in childhood represent an important and potentially at-risk population to conduct research with regard to the long-term effects of childhood adversity.

The present pilot study investigated the potential impact of exposure to childhood adversity on variables known to be related to posttraumatic stress (*e.g.*, attachment, mental health, and perceived stress) in a clinic sample of pregnant women. Specifically, the study seeks to answer the following questions:

1. Are there differences, based on exposure to ACEs, in mother's recollections and perceptions of her own childhood experiences of being parented?
2. Do differences exist, based on exposure to ACEs, in the overall quality (*e.g.*, health or lack thereof) of maternal/fetal attachment?
3. Are relationships present between exposure to ACEs and maternal mental health outcomes?
4. Is there a relationship between exposure to ACEs and perceived maternal stress?
5. Are there interrelationships between ACEs, perception of parenting, maternal/fetal attachment, mental health, and perceived stress?

**FIG. 1.**
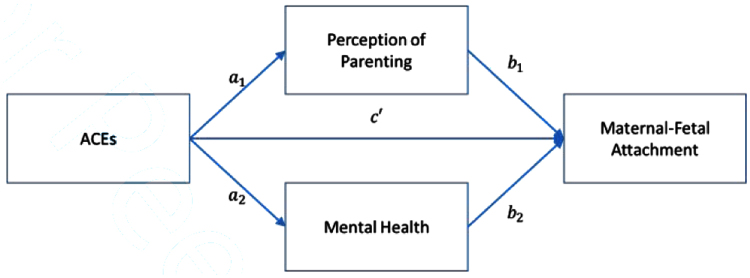
Theoretical model.

We hypothesize an additive effect of ACEs as they impact mother's recollections and perceptions of her own early childhood experiences of being parented. Specifically, it is predicted that ACEs will be associated with poor retrospective bonding reports (path 1 in theoretical model shown in Fig. 1) on the Parental Bonding Instrument (PBI). It is also hypothesized that an additive effect of ACEs will occur in relation to current maternal/fetal attachment, evidenced by low scores on the Maternal Fetal Attachment Scale (MFAS) (path 2). It is hypothesized that ACEs will be associated with higher symptoms of posttraumatic stress disorder (PTSD) on the PTSD Checklist for DSM 5 (PCL-5) and general psychiatric distress (path 3) on the Symptom Checklist (SCL-27). Lastly, it is hypothesized that two meditational effects will be found: first, an indirect effect of perceptions of parenting on the relationship between ACEs and maternal/fetal attachment is expected (path a) and second, it is expected that mental health symptom severity will mediate the relationship between ACEs and maternal/fetal attachment (path b).

## Materials and Methods

### Participants

Participants consisted of 101 pregnant women in their third trimester recruited from the Virginia Commonwealth University Health System (VCUHS) Department of Obstetrics and Gynecology in Richmond. Patients were recruited during routine, scheduled, obstetric follow-up appointments. The eligibility criteria included being at least 18 years of age, pregnant in the third trimester, English speaking, and ability to provide informed consent. This study has been approved by the VCU Institutional Review Board (IRB HMO8206). 

### Procedure

Interested potential participants completed an initial online screening *via* a secure online survey platform to determine eligibility. An explanation of procedures was provided and informed consent obtained. Each participant was asked to complete a self-report battery of online questionnaires pertaining to stressful life events, relationships with primary caregivers, pregnancy, mental health, and perceived stress. Participants could elect not to answer any questions, and could opt to end the survey at any time. At the completion of the questionnaires, referral information for mental health support was provided to all participants. Participants who completed the study were compensated for their time and effort with a $20 Walmart gift card.

### Measures

The ACE Study questionnaire^1^ is a 10-item scale that was adapted and can be used to retrospectively assess forms of abuse, neglect, and household dysfunction. Scores range from 0 to 10, with the latter representing full exposure at some point in the first 18 years of life, to all 10 forms of household dysfunction and abuse detailed in the questionnaire.

The PBI^10^ is a 25-item instrument that assesses the quality of the parent/child relationship during childhood. Responses are scored on a 4-point Likert scale and subscale scores are assigned corresponding to different bonding categories (*e.g.*, optimal parenting, affectionate constraint, affectionless control, and neglectful parenting). Optimal bonding is characterized by high levels of caring and low overprotection.

The MFAS^11^ is a 24-item Likert scale designed to measure the construct of maternal/fetal attachment during pregnancy. The instrument has five subscales that propose to measure aspects of the relationship between mother and fetus, which include the extent to which women engage in behaviors that represent affiliation and interaction with their unborn baby.

The PCL-5^12^ is a 20-item self-report measure that assesses 20 DSM-5 symptoms of PTSD. The PCL-5 can be used to screen individuals for PTSD and to make a provisional diagnosis of PTSD.

The SCL-27-plus is a short, multidimensional screening instrument for mental health problems. It contains five scales on current symptoms: depressive, vegetative, agoraphobic, and sociophobic symptoms and pain; a global severity index (GSI-27); a lifetime assessment for depressive symptoms; and a screening question for suicidality.

The Perceived Stress Scale is the most widely used psychological instrument for measuring the perception of stress. It is the measure to the degree of situations in one's life appraised as stressful. Items include 10 questions that were designed to tap how unpredictable, uncontrollable, and overloaded respondents find their lives. The scale also includes a number of direct queries about current levels of experienced stress.

### Statistical analyses

Path analysis using full information maximum likelihood estimation was used to fit the theoretical model described in Figure 1. Errors terms for each of the mediator variables were allowed to correlate to account for unmeasured factors that may affect the mediators. The addition of correlated errors leads to a saturated model so no model fit statistics is available or reported. In addition, to account for demographic characteristics, participant age and race (African American vs white) were included as a predictor for each endogenous variable. To test for potential indirect effects, percentile bootstrap confidence intervals were used as this method. This method for testing the indirect effect provides adequate power while also maintaining type I error rates to the nominal level.^13^ Each statistical test was performed using an alpha of 0.05. The lavaan package^14^ in the R statistical software was used for all analyses.

## Results

Descriptive statistics for the sample are provided in Table 1. Overall, the majority of the sample was white (67.3%) and married (61.3%). The sample was heterogeneous with regard to education level and income.

**Table 1.** Demographic Characteristics

Variable	Mean (SD) or N (%)
Age	29.2 (5.45)
Race	
White	68 (67.3%)
African American	27 (26.7%)
Native American	2 (2.0%)
Pacific Islander	4 (4.0%)
Other/unsure	4 (4.0%)
Ethnicity	
Hispanic	5 (5.0%)
Weeks pregnant	33.1 (3.32)
Relationship status	
Married	76 (61.3%)
Separated	1 (0.8%)
Cohabitating	7 (5.6%)
In a relationship	18 (14.5%)
Single	7 (5.6%)
Education	
Some high school	4 (3.2%)
High school graduate	11 (8.9%)
Some college	29 (23.4%)
Four-year college graduate	26 (21.0%)
Some graduate school	4 (3.2%)
Graduate degree	34 (27.4%)
Income	
Less than $10,000	16 (15.8%)
$10,000 to less than $20,000	14 (13.9%)
$20,000 to less than $40,000	17 (16.8%)
$40,000 to less than $60,000	20 (19.8%)
$60,000 to less than $80,000	9 (8.9%)
$80,000 to less than $100,000	4 (4.0%)
$100,000 to less than $200,000	8 (7.9%)
More than $200,000	1 (1.0%)
Employment status	
Full-time	51 (50.5%)
Part-time	18 (17.8%)
Not employed	26 (25.7%)
Student	6 (5.9%)
ACE score	1.7 (2.05)
Mother PBI care score	26.5 (9.68)
Mother PBI overprotection score	14.9 (8.76)
Father PBI care score	25.6 (10.73)
Father PBI overprotection score	12.4 (9.01)
MFAS score	87.7 (10.03)
PCL score	14.9 (16.8)
SCL score	15.7 (17.32)
PSS score	13.2 (6.64)

ACE, adverse childhood experience; MFAS, Maternal Fetal Attachment Scale; PBI, Parental Bonding Instrument; PSS, Perceived Stress Scale; PCL, PTSD Checklist; PTSD, posttraumatic stress disorder; SCL, Symptom Checklist.

Exposure to adverse childhood events was low with a mean of 1.7 out of a maximum of 10. ACE scores were normally distributed. In addition, participants were, on average, high on retrospective reports of viewing their own parents as caring, low on retrospective reports of regarding parents as intrusive and controlling, and scored generally high with regard to maternal/fetal attachment scores.

Results from the path analysis are provided in Table 2. As predicted, increased exposure to adverse childhood events was negatively associated with retrospective report of viewing one's mother and father as caring and involved (<0.001). ACE exposure was also a statistically significant predictor of viewing one's mother and father as intrusive and controlling (<0.001). Similarly, aligned with our hypotheses, increased exposure to ACEs were positively associated with self-reported PTSD symptoms, depressive and anxious symptomatology, and perceived stress. These results were also statistically significant.

**Table 2.** Unstandardized Estimates of Mediation Pathways from Adverse Childhood Events to Maternal/Fetal Attachment

Predictor	Outcome		
	Estimate	SE	p
Mother care score			
ACE score	−3.192	0.342	<0.001
Age	0.156	0.143	0.274
Race (African American)	2.812	1.741	0.106
Mother overprotection score			
ACE score	1.505	0.401	<0.001
Age	0.080	0.157	0.610
Race (African American)	−3.086	1.953	0.114
Father care score			
ACE score	−2.723	0.424	<0.001
Age	0.143	0.186	0.443
Race (African American)	2.096	2.439	0.390
Father overprotection score			
ACE score	1.222	0.432	0.005
Age	0.320	0.175	0.068
Race (African American)	−3.547	2.333	0.128
PCL score			
ACE score	5.139	0.611	<0.001
Age	−0.508	0.248	0.040
Race (African American)	1.566	3.078	0.611
SCL score			
ACE score	5.425	0.610	<0.001
Age	−0.233	0.250	0.350
Race (African American)	2.935	3.089	0.342
PSS score			
ACE score	1.671	0.260	<0.001
Age	0.046	0.107	0.669
Race (African American)	3.977	1.335	0.003
MFAS score			
ACE score	0.453	0.758	0.550
Mother care score	−0.202	0.143	0.160
Mother overprotection score	−0.169	0.140	0.225
Father care score	0.377	0.116	0.001
Father overprotection score	−0.052	0.138	0.705
PCL score	0.173	0.134	0.195
SCL score	−0.157	0.123	0.201
PSS score	−0.308	0.239	0.196
Age	0.424	0.181	0.019
Race (African American)	−3.202	2.275	0.159

No direct effect of adverse childhood events on maternal/fetal attachment was found accounting for the seven potential mediators.

Viewing one's own father as caring was positively associated with maternal/fetal attachment (*p* = 0.001). In addition, age was positively associated with maternal/fetal attachment (*p* = 0.019). No other statistically significant direct effects on maternal/fetal attachment were found.

The indirect effect of ACEs on maternal/fetal attachment through perceiving one's father as caring was statistically significant and negative (Table 3). This indicates that part of the negative effect of ACEs on attachment can be explained by ACEs being associated with lower father caring. No other statistically significant indirect effects were observed.

**Table 3.** Estimates of Indirect Effects of Adverse Childhood Experiences on Maternal/Fetal Attachment with 95% Percentile Bootstrap Confidence Intervals

Mediator	Estimate	Lower	Upper
Mother caring	−0.643	1.703	0.456
Mother overprotection	0.255	−0.162	0.715
Father caring	1.028	0.258	2.351
Father overprotection	0.064	−0.425	0.666
PTSD	0.891	−2.348	0.976
Mental health	−0.853	−0.874	2.182
Stress	−0.515	−0.387	1.513

## Discussion

The present pilot study investigated the potential impact of exposure to ACEs on maternal outcome variables. Increased exposure to ACEs was associated with self-reported post-traumatic stress, depression, and anxiety symptoms, as well as perceived stress. These results are consistent with the converging and growing evidence linking childhood adversity with numerous long-term pernicious effects in adulthood, including risk for current and lifetime PTSD,^15^ chronic depression,^16^ severity of depressive course trajectory,^17^ substance use disorders, eating disorders, and borderline personality disorder.

Our study had several limitations. This sample was unique in terms of the low mean ACE score of 1.7. It would have been helpful if our sample had a greater range of ACE scores to assess outcomes, and it is possible that the low scores biased the results. Our sample was also mostly white, educated, and married, which is also not representative of the U.S. population. It is also possible that recruiting patients during routinely scheduled obstetric follow-up inadvertently self-selected for a more homogeneous racial sample. This study relied upon assessment of events and symptoms in a single point in time. Although this was the most feasible methodology for the purposes of this pilot study with a small sample size, it is neither ideal nor sufficient to capture the complexity of the long-term impact of exposure to ACEs, which necessitates prospective data. In addition, the inherent biases involved in the subjective nature of self-report data utilized may also have contributed to these results. Assessing exposure to traumatic events and psychiatric symptomatology *via* structured or semistructured clinical interviews would likely yield more valid and reliable results. We also do not know if pregnancy could have affected the results since our outcome measures have not been validated during pregnancy. Finally, the study design does not allow for definite conclusions about causal effects because unmeasured factors may account for observed associations.

No direct effect of adverse childhood events on maternal/fetal attachment was found. Potential explanations for our results include the methodology limitations described above with regard to self-report data. Future research examining childhood adversity and attachment would benefit from utilizing “gold standard” structured clinical interviews such as the Adult Attachment Interview, Parent Development Interview, and Strange Situation Procedure (postnatally).

Viewing one's father as caring was positively associated with maternal/fetal attachment. Relatedly, the indirect effect of ACEs on maternal/fetal attachment through perceived father caring was statistically significant. These results are consistent with previous research that provides support for the notion of distinct effects for child/father relationships^18^ that may persist across development.

The present findings provide an example of the descriptive information that can be gained by pilot study efforts to integrate developmental science into a clinic setting. It may be useful for health providers to consider incorporating the ACE questionnaire to identify exposure to childhood adversity and events that may increase an individual's risk for toxic stress and negative health outcomes. Future prospective research is needed to examine the long-term effects of exposure to childhood adversity with expectant mothers, and results can then be translated into psychotherapy interventions to optimally support their psychological functioning in preparation for motherhood.

## Abbreviations Used

ACE: adverse childhood experiences
MFAS: Maternal Fetal Attachment Scale
PBI: Parental Bonding Instrument
PCL-5: PTSD Checklist for DSM 5
PSS: Perceived Stress Scale
PTSD: posttraumatic stress disorder
SCL-27: Symptom Checklist
